# ﻿Four new species of *Erioscyphella* (Leotiomycetes, Helotiales) from southwestern China

**DOI:** 10.3897/mycokeys.114.138647

**Published:** 2025-02-14

**Authors:** Le Luo, Kandawatte W. Thilini Chethana, Qi Zhao, Hong-Li Su, Cui-Jin-Yi Li, Vinodhini Thiyagaraja, Fatimah Al-Otibi, Kevin D. Hyde

**Affiliations:** 1 Key Laboratory of Phytochemistry and Natural Medicines, Kunming Institute of Botany, Chinese Academy of Sciences, Kunming 650201, Yunnan, China; 2 Center of Excellence in Fungal Research, Mae Fah Luang University, Chiang Rai 57100, Thailand; 3 School of Science, Mae Fah Luang University, Chiang Rai 57100, Thailand; 4 Guiyang Institute of Humanities and Technology, Guiyang 550025, Guizhou, China; 5 Department of Botany and Microbiology, College of Science, King Saud University, P.O. Box 22452, Riyadh 11495, Saudi Arabia

**Keywords:** 4 novel species, Lachnaceae, morphology, phylogeny, taxonomy

## Abstract

*Erioscyphella* is found across various regions and is part of the family Lachnaceae (Helotiales). It is distinguished by its white to orange disc-shaped apothecia, white to brown receptacles, and granulated hairs that contain amorphous or resinous material. These hairs lack swelling apices and crystals. Additionally, this genus is unique for its long ascospores. In the present study, we collected eight specimens from southwestern China. Morphological and phylogenetic analyses based on the combined LSU, ITS, mtSSU and *RPB2* dataset showed that our specimens represent four new species of *Erioscyphella*, including *E.ailaoensis*, *E.baimana*, *E.gelangheica* and *E.tengyueica*. Here, we provide complete morphological descriptions with illustrations and sequence data essential for future taxonomic and evolutionary research.

## ﻿Introduction

The monophyletic genus *Erioscyphella* belongs to the family Lachnaceae (Dennis 1954; Spooner 1987; Cantrell and Haines 1997; Guatimosim et al. 2016; Tochihara and Hosoya 2022; Wijayawardene et al. 2020, 2022; Hyde et al. 2024) and includes 22 records (Index Fungorum 2024). The original description of *Erioscyphella* by Kirschstein (1939), which lacked typification, was inaccurately characterized based on traits that lack taxonomic significance, such as filiform, colored, and pigmented ascospores, as well as lanceolate paraphyses (Korf 1978; Perić and Baral 2014). It was not until Haines (1984) selected *E.longispora*, under which *Pezizaabnormis* was later synonymized, as the lectotype of *Erioscyphella* that a more accurate characterization was established. These features are now recognized as insufficient for distinguishing this genus from related taxa. This genus was initially confused with the closely related genus *Lachnum*, but detailed morphological and molecular phylogenetic studies based on LSU, ITS, mtSSU and *RPB2* data have since clarified their distinct characteristics and proposed a new concept based on the examination of Japanese materials (Tochihara and Hosoya 2022). *Erioscyphella* is characterized by scattered and cupulate apothecia on leaves or bamboo sheaths, straight or irregularly curved, septate and granulated hairs, mostly covered by apical amorphous materials or resinous material, lanceolate or filiform paraphyses, 8-spored asci with an amyloid apical pore and fusiform to long needle-like ascospores (Kirschstein 1939; Perić and Baral 2014; Tochihara and Hosoya 2022). There are no reports of asexual morph in this genus.

The species of *Erioscyphella* are primarily distributed in temperate and tropical regions, commonly inhabiting decaying wood and plant debris. *Erioscyphella* species are distributed mainly in China and a few other countries; *E.curvispora* is described from Montenegro (Perić and Baral 2014), *E.griseibambusicola*, *E.latispora*, *E.lunata*, *E.lushanensis* and *E.subinsulae* are collected from China (Guatimosim et al. 2016; Tello and Baral 2016; Li et al. 2022; Su et al. 2023); *E.euterpes* collected from Puerto Rico (Guatimosim et al. 2016). In contrast to the above-mentioned species that show a narrower distribution, *E.abnormis* shows a worldwide distribution, especially endemic in tropical regions (Tello and Baral 2016). They play a crucial role in breaking down organic matter, thereby aiding in nutrient cycling within their ecosystems. The ecological role of *Erioscyphella* as decomposers underscores their importance in forest ecosystems (Tochihara and Hosoya 2022; Niego et al. 2023a, 2023b). Ongoing research aims to better define species boundaries and elucidate the phylogenetic relationships within the genus through morphological studies and DNA sequencing. This work aids in the precise classification and discovery of new species.

During the investigation of Leotiomycetes in southwest China (Li et al. 2022, 2024a, 2024b; Su et al. 2022, 2023; Luo et al. 2024; Thiyagaraja et al. 2024), eight collections of *Erioscyphella* were obtained. We used morphological and phylogenetic analyses based on LSU, ITS, mtSSU and *RPB2* data to confirm that these eight collections differ from all known species of *Erioscyphella*. We introduce four species to accommodate these collections. Here, we provide complete morphologies, illustrations, and their phylogenetic relationships for future taxonomic and evolutionary studies.

## ﻿Material and methods

### ﻿Specimen collection and morphological examination

We collected eight specimens from southwest China. All samples were collected from highly humid, natural broadleaf forests and protected areas with minimal human access. Altitudes were determined by the GPS device. The fruiting bodies were discovered on the surface of extremely wet, decaying wood litter. The samples were dehydrated in a dehydrator at a temperature range of 25–30 °C. After studying the morphology of the specimens and getting their genomic DNA, they were deposited at the Cryptogamic Herbarium of the Kunming Institute of Botany, Chinese Academy of Sciences (KUN-HKAS). Facesoffungi and Index Fungorum numbers were obtained as in Jayasiri et al. (2015) and Index Fungorum (2024), respectively. All the species identifications followed Chethana et al. (2021). The morphological descriptions were submitted to the Greater Mekong Subregion database (Chaiwan et al. 2021). The dried specimens were examined with a stereomicroscope (C-PSN, Nikon, Japan) and were captured with a digital camera (Canon EOS 70D, Japan) connected to the stereomicroscope. Free-hand sections of the dried specimens were mounted in a drop of water for observing microscopic characteristics, such as apothecia, exciple, paraphyses, asci and ascospores, using a Nikon compound microscope (Nikon, Japan) equipped with a DS-Ri2 camera. In addition, the sections were pretreated with Melzer’s reagent for the Iodine test (MLZ) (Tochihara and Hosoya 2022). Microstructures were measured using the Tarosoft (R) Image Frame Work program v.0.97 (Tarosoft, Thailand). The obtained measurements were presented in the format of (a–) b–c(–d), where ‘a’ represented the minimum value, ‘d’ represented the maximum value, and the range ‘b–c’ reflected the 90% confidence interval. The x̄ indicated the average value of measurements. Ascospore measurements were given as [n/m/p], indicating that the n number of ascospores were measured from m ascomata of the p number of collections. Images used for figures were processed with Adobe Photoshop CS6 Extended version 13.0 × 64 (Adobe Systems, USA).

### ﻿DNA extraction, PCR amplifications and sequencing

Genomic DNA was extracted from the dried apothecia (around 50–100 mg) using a TSP101 DNA extraction kit (TSINGKE, China). Following the latest studies (Tochihara and Hosoya 2022; Su et al. 2023; Luo et al. 2024), LSU, ITS, mtSSU and *RPB2* were used for PCR amplification, using the primers LR0R/LR5 (Vilgalys and Hester 1990), ITS1F/ITS4 (White et al. 1990; Gardes and Bruns 1993), mrSSU1/mrSSU3R (Zoller et al. 1999) and f*RPB2*-5F/f*RPB2*-7cR (Liu et al. 1999), respectively. For LSU, ITS, mtSSU and *RPB2*, the total volume of PCR amplifications was 25 μL, comprising 12.5 μL 2 × PCR G013 Taq MasterMix with Dye (Applied Biological Materials, Canada), 1 μL of each primer (10 μM), 2 μL genomic DNA, and 8.5 μL of sterilized, distilled water. Amplifications of LSU, ITS and *RPB*2 were conducted under the following conditions: pre-denaturation at 95 °C for 5 min, followed by 35 cycles of denaturation at 95 °C for 20 sec, annealing at 56 °C (LSU)/53 °C (ITS and *RPB*2) for 10 sec, elongation at 72 °C for 20 sec, and final elongation at 72 °C for 7 min. For mtSSU, the total volume of PCR amplifications was 25 μL, which comprised 21 μL 1 × PCR TSE101 Mix (TSINGKE, China), 1 μL of each primer (10 μM), and 2 μL genomic DNA. Amplifications of mtSSU were conducted under the following conditions: initial denaturation at 98 °C for 3 min, followed by 40 cycles of denaturation at 98 °C for 1 min, annealing at 52 °C for 1 min, elongation at 72 °C for 1 min, and final elongation at 72 °C for 10 min. Gel electrophoresis with 1% TAE and TSJ003 GoldView nucleic acid dye (TSINGKE, China) was used to confirm the obtained PCR products. Finally, the PCR products were sequenced at the Tsingke Biotechnology Co., Ltd., Kunming, China. Newly produced sequences were deposited in the GenBank and the accession numbers were given in Table 1.

**Table 1.** Taxa included in the phylogenetic analyses and the GenBank accession numbers of LSU, ITS, mtSSU and *RPB2* sequences.

Species	Strain	Gene accession No.				References
		ITS	LSU	mtSSU	*RPB2*	
*Capitotrichabicolor*	TNS-F-65670	LC424834	LC424942	LC533244	LC425011	Tochihara and Hosoya (2022)
*Capitotricharubi*	TNS-F-65752	LC438560	LC438573	LC533243	LC440395	Tochihara and Hosoya (2022)
*Erioscyphellaabnormis*	TNS-F-16609	AB705234	LC533175	LC533256	LC533184	Tochihara and Hosoya (2022)
*Erioscyphellaabnormis*	TNSF38452	LC669457	LC533171	LC533262	LC533210	Tochihara and Hosoya (2022)
*Erioscyphellaabnormis*	TNS-F-80478	LC424837	LC424949	LC533283	-	Tochihara and Hosoya (2019)
*Erioscyphellaabnormis*	TNS-F-46841	LC669474	LC533170	LC533279	LC533209	Tochihara and Hosoya (2022)
***Erioscyphellaailaoensis***	**HKAS135686** ^(T)^	**PQ349783**	**PQ349775**	**PQ358800**	**PQ424108**	This study
***Erioscyphellaailaoensis***	**HKAS135687**	**PQ349784**	**PQ349776**	**PQ358801**	**PQ424109**	This study
*Erioscyphellaalba*	MFLU16-0614^(T)^	MK584965	MK591990	-	-	Ekanayaka et al. (2019)
*Erioscyphellaaseptata*	MFLU16-0590^(T)^	MK584957	MK591986	-	MK388223	Ekanayaka et al. (2019)
***Erioscyphellabaimana***	**HKAS135697** ^(T)^	**PQ349785**	**PQ349777**	**PQ358802**	**PQ424110**	This study
***Erioscyphellabaimana***	**HKAS135696**	**PQ349786**	**PQ349778**	**PQ358803**	**PQ424111**	This study
*Erioscyphellaboninensis*	TNS-F-26520^(T)^	NR185389	LC533151	LC533254	LC533196	Tochihara and Hosoya (2022)
*Erioscyphellabrasiliensis*	MFLU16-0577b	MK584967	MK591993	-	-	Ekanayaka et al. (2019)
*Erioscyphellabrasiliensis*	TNS-F-46419	LC669456	LC533133	LC533278	LC549672	Tochihara and Hosoya (2022)
*Erioscyphellacurvispora*	KL 381^(T)^	MH190414	MH190415	-	-	Perić and Baral (2014)
*Erioscyphellaeuterpes*	PR 147	U58640	-	-	-	Cantrell and Haines (1997)
*Erioscyphellafusiforme*	MFLU15-0230^(T)^	MK584948	MK591975	-	MK614728	Ekanayaka et al. (2019)
***Erioscyphellagelangheica***	**HKAS135689** ^(T)^	**PQ349787**	**PQ349779**	**PQ358804**	-	This study
***Erioscyphellagelangheica***	**HKAS135695**	**PQ349788**	**PQ349780**	**PQ358805**	-	This study
*Erioscyphellagriseibambusicola*	HKAS124657	OP451797	OP451791	OP451844	OP432252	Su et al. (2023)
*Erioscyphellagriseibambusicola*	HKAS124656^(T)^	OP451796	OP451790	OP451843	OP432251	Su et al. (2023)
*Erioscyphellahainanensis*	TNS-F-35056	LC669465	LC533169	LC533275	LC533206	Tochihara and Hosoya (2022)
*Erioscyphellahainanensis*	TNS-F-35049	LC669452	LC533168	LC533274	LC533205	Tochihara and Hosoya (2022)
*Erioscyphellainsulae*	TNS-F-26500	LC669448	LC533149	LC533252	LC533194	Tochihara and Hosoya (2022)
*Erioscyphellainsulae*	TNS-F-39720^(T)^	LC669451	LC533177	LC533261	LC533207	Tochihara and Hosoya (2022)
*Erioscyphellalatispora*	HKAS124391	OP113849	OP113850	-	OP715727	Li et al. (2022)
*Erioscyphellalatispora*	HKAS124389^(T)^	OP310823	OP113844	-	OP715728	Li et al. (2022)
*Erioscyphellalunata*	JA-CUSSTA 8292	KX501132	KX501133	-	-	Tello and Baral (2016)
*Erioscyphellalushanensis*	HMAS81575	JF937582	-	-	-	Zhao and Zhuang (2011)
*Erioscyphellaotanii*	TNS-F-81472^(T)^	NR185393	LC533179	LC533286	LC533226	Tochihara and Hosoya (2022)
*Erioscyphellapapillaris*	TNS-F-81272^(T)^	NR185391	LC533161	LC533285	LC533204	Tochihara and Hosoya (2022)
*Erioscyphellaparalushanensis*	TNS-F-61920^(T)^	NR185390	LC533141	LC533267	LC533220	Tochihara and Hosoya (2022)
*Erioscyphellasasibrevispora*	TNS-F-80399	LC669470	LC533173	LC533268	LC533216	Tochihara and Hosoya (2022)
*Erioscyphellasasibrevispora*	TNS-F-81401^(T)^	LC669472	LC533174	LC533269	LC533217	Tochihara and Hosoya (2022)
*Erioscyphellasclerotii*	TNS-F-26492	LC669438	LC533152	LC533255	LC533197	Tochihara and Hosoya (2022)
*Erioscyphellasclerotii*	TNS-F-38480	LC669458	LC533134	LC533263	LC549673	Tochihara and Hosoya (2022)
*Erioscyphellasclerotii*	MFLU 16-0569	MK584951	MK591980	-	-	Ekanayaka et al. (2019)
*Erioscyphellasclerotii*	MFLU 18-0688	MK584969	MK591995	-	-	Ekanayaka et al. (2019)
*Erioscyphellasinensis*	TNS-F-32161	LC669449	LC533167	LC533273	LC533219	Tochihara and Hosoya (2022)
*Erioscyphellasinensis*	TNS-F-16838	AB481280	LC533164	LC533235	AB481364	Tochihara and Hosoya (2022)
*Erioscyphellasubinsulae*	HKAS 124659	OP451799	OP451793	OP451846	OP432254	Su et al. (2023)
*Erioscyphellasubinsulae*	HKAS 124660	OP451800	OP451794	OP451847	OP432255	Su et al. (2023)
*Erioscyphellasubinsulae*	HKAS 124661	OP451801	OP451795	OP451848	OP432256	Su et al. (2023)
*Erioscyphellasubinsulae*	HKAS 124658^(T)^	OP451798	OP451792	OP451845	OP432253	Su et al. (2023)
***Erioscyphellatengyueica***	**HKAS135688** ^(T)^	**PQ349789**	**PQ349781**	**PQ358806**	-	This study
***Erioscyphellatengyueica***	**HKAS135693**	**PQ349790**	**PQ349782**	**PQ358807**	-	This study
*Lachnellulacalyciformis*	TNS-F-81248	LC438561	LC438574	LC533247	LC438590	Tochihara and Hosoya (2022)
*Lachnellulasuecica*	TNS-F-16529	AB481248	LC424944	LC533231	AB481341	Tochihara and Hosoya (2022)
*Neodasyscyphacerina*	TNS-F-65625	LC424836	LC424948	LC533242	LC425013	Tochihara and Hosoya (2022)

*Names in indicate the specimens from the current study. Names with ^(T)^ indicate type specimens and - denotes unavailable data in the GenBank.

### ﻿Phylogenetic analyses

New DNA sequences generated from forward and reverse primers were assembled using BioEdit v.7.2.5 (Hall 1999) to obtain consensus sequences. The concatenated sequences were used to search for the closer relatives in the NCBI (Johnson et al. 2008). According to the close relatives and recent studies, the newly generated sequences and some published sequences were used for the phylogenetic analyses (Table 1). *Capitotrichabicolor* (TNS-F-65670), *C.rubi* (TNS-F-65752), *Lachnellulacalyciformis* (TNS-F-81248), *L.suecica* (TNS-F-16529) and *Neodasyscyphacerina* (TNS-F-65625) were used as the outgroup taxa. The phylogenetic analysis was conducted based on the datasets including reference DNA sequences and newly generated DNA sequences using OFPT (Zeng et al. 2023) with the following protocol. Datasets of each gene region were first independently aligned with ‘auto’ strategy (based on data size) by MAFFT (Katoh and Standley 2013) and trimmed with ‘gappyout’ method (based on gaps’ distribution) by TrimAl (Capella-Gutiérrez et al. 2009). The best-fit nucleotide substitution model for each dataset was then selected based on the Bayesian information criterion (BIC) from twenty-two common DNA substitution models with rate heterogeneity by ModelFinder (Kalyaanamoorthy et al. 2017). Afterwards, all datasets were concatenated with partition information for the subsequent phylogenetic analyses. Maximum likelihood with 1000 replicates was performed using ultrafast bootstrap approximation (Hoang et al. 2018) with SH-like approximate likelihood ratio test (SH-aLRT) (Guindon et al. 2010) in IQ-TREE (Nguyen et al. 2015). The consensus tree was summarized based on the extended majority rule. Bayesian inference (BI) analyses were run in the CIPRES Science Gateway v.3.3 (Miller et al. 2010). The best-fit nucleotide substitution models were determined using jModelTest2 on XSEDE (2.1.6). The BI was performed in MrBayes on XSEDE v. 3.2.7a (Ronquist et al. 2012), with four simultaneous Markov chain Monte Carlo (MCMC) chains and four runs for 3,000,000 generations, with trees sampled at each 300^th^ generation. The first 25% of trees were discarded as burn-in, and BI posterior probabilities (PP) were conducted from the remaining trees. The consensus phylograms were visualized on FigTree v. 1.4.4, and edited with Adobe Illustrator CC 2019, Adobe Systems (USA). Decisions as to whether species are new followed the polyphasic approach as recommended by Chethana et al. (2021) and Maharachchikumbura et al. (2021).

## ﻿Results

### ﻿Phylogenetic analysis

**Figure 1.**
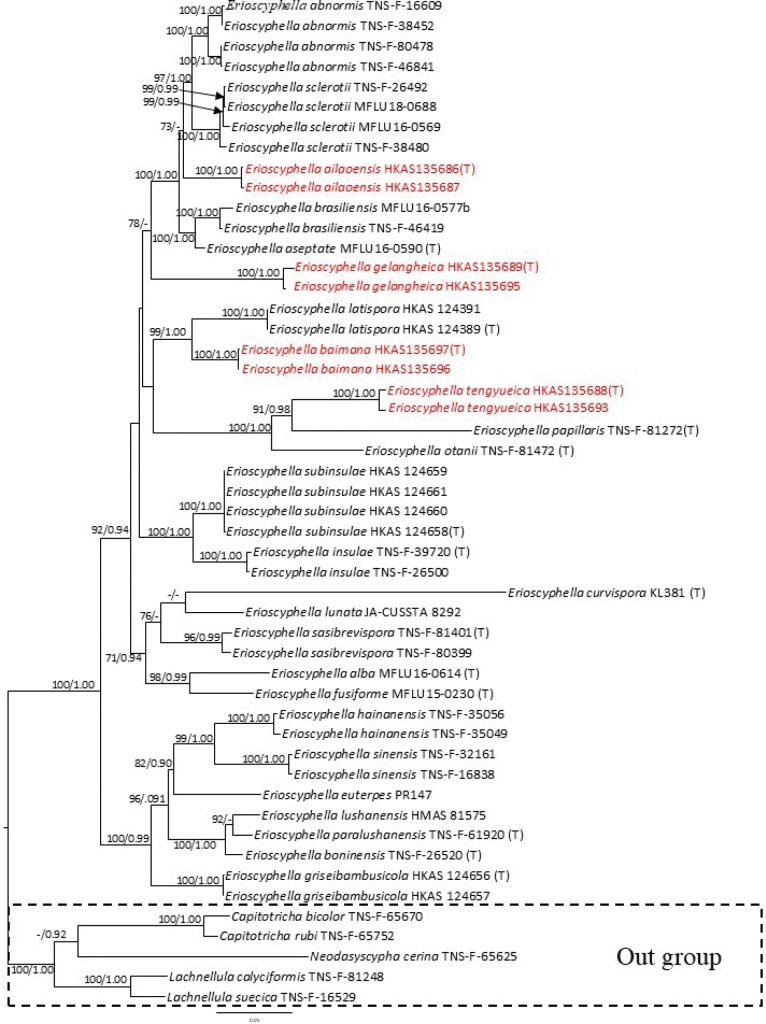
The Maximum Likelihood tree based on the combined LSU, ITS, mtSSU and *RPB*2 sequence data for *Erioscyphella*. *Capitotrichabicolor* (TNS-F-65670), *C.rubi* (TNS-F-65752), *Lachnellulacalyciformis* (TNS-F-81248), *L.suecica* (TNS-F-16529) and *Neodasyscyphacerina* (TNS-F-65625) are used as the outgroup taxa. The MLBP ≥ 70% and BPP ≥ 0.90 are shown at the nodes as MLBP/BPP. MLBS < 70% and BPP < 0.90 are expressed as a hyphen (“-”). Names with (T) indicate type specimens. Names in red indicate new species.

The phylogenetic analyses were based on 45 *Erioscyphella* taxa, including *C.rubi* (TNS-F-65752), *C.bicolor* (TNS-F-65670), *L.calyciformis* (TNS-F-81248), *L.suecica* (TNS-F-16529) and *N.cerina* (TNS-F- 65625) as the outgroup taxa. The alignment comprised 4 partitions and 3944 total sites (ITS: 758 bp; LSU: 1105 bp; mtSSU: 947 bp; *RPB*2: 1134 bp), with 14.359% gaps and completely undetermined characters. The ML tree has the same topology as the BI tree. The best ML tree with a final optimization likelihood of -25316.078809 is displayed in Fig. 1. In the BI analyses, the final average standard deviation of split frequencies was 0.007666, which revealed convergence. In the multi-gene phylogenetic tree based on the combined ITS, LSU, mtSSU and *RPB*2 dataset, all taxa of *Erioscyphella* clustered together. *Erioscyphellaailaoensis* was sister to the clade comprising *E.sclerotii* and *E.abnormis*, with 73% maximum likelihood bootstrap (MLBP) and 0.89 Bayesian posterior probabilities (BPP) support. *Erioscyphellagelangheica* was sister to the clade comprising *E.sclerotii* and *E.abnormis*, *E.ailaoensis*, *E.brasiliensis* and *E.aseptata* with 78% MLBP and 0.88 BPP support. *Erioscyphellabaimana* was sister to *E.latispora* with 99% MLBP and 1.00 BPP support. Besides, *E.tengyueica* was sister to *E.papillaris* with 91% MLBP and 0.98 BPP. The phylogenetic result showed that *Erioscyphella* species clustered together, similar to previously published studies (Su et al. 2023).

### ﻿Taxonomy

#### ﻿ Erioscyphella ailaoensis

Taxon classificationFungiHelotialesLachnaceae

L. Luo, K.D. Hyde & H.L. Su sp. nov.

E5DEFE74-5F9A-51F3-B1D4-E15C29F9F1AD

Index Fungorum: IF902529

Facesoffungi Number: FoF16389

Fig. 2

##### Etymology.

The epithet “*ailaoensis*” refers to the collection site, Ailao Mountain, where the holotype specimen was collected.

##### Holotype.

HKAS135686.

##### Description.

***Saprobic*** on the dead bark. ***Sexual morph*: *Apothecia*** scattered to partly gregarious, superficial, 1–2.4 mm in diameter, 0.4–1.4 mm high when dry, discoid to cupulate, shortly stipitate, externally covered with short, white to brown hairs. ***Disc*** concave, surface slightly rough, yellow to brown. Margin flat to slightly involute, pale yellow, covered with yellow to pale brown hairs. ***Receptacle*** discoid to cupulate, yellow to pale brown, clothed entirely with short, yellow to pale brown hairs. ***Stipe*** 0.2–1 mm in diameter, 0.2–0.6 mm long when dry, cylindrical, solitary, yellow to pale brown, clothed with yellow to pale brown hairs. ***Hairs*** 22–92 × 3.0–4.3 µm (x̄ = 51 × 3.7 µm, n = 30), clavate to cylindrical, straight to slightly curved, septate, hyaline, thick-walled, covered with hyaline granules, obtuse apex, apical amorphous or resinous material. ***Hymenium*** 120–230 µm (x̄ = 153 µm, n = 12), concave, surface slightly rough, light yellowish brown in dry. ***Medullary excipulum*** 40–90 µm (x̄ = 58 µm, n = 20), thin, hyaline to light yellow, thin-walled, smooth cells of ***textura oblita***, 2.1–5.9 µm (x̄ = 3.7 µm, n = 50) in diameter. ***Ectal excipulum*** 55–80 µm (x̄ = 65 µm, n = 20), thin-walled, smooth, light yellowish cells of ***textura prismatica*** to ***globulosa***, 2–4.9 µm (x̄ = 3.0 µm, n = 60) in diameter. ***Paraphyses*** 100–138 × 1.6–3.9 µm (x̄ = 117 × 2.8 µm, n = 25), longer than asci, filiform, straight to slightly curved, aseptate, hyaline, thin-walled, rough, with slightly acute apex. ***Asci*** 85–143 × (4.5–) 5.5–9.0(–9.5) µm (x̄ = 100 × 7.3 µm, n = 34), 8-spored, unitunicate, overlapping fascicles, clavate, straight to slightly curved, inoperculate, hyaline, apically thickened wall, laterally relatively thin, slightly smooth, with an apical, non-amyloid pore and tapered ends, J- in MLZ. ***Ascospores*** (50/14/2) (43.0–)45.5–97(–101.0) × 1.4–2.4(–2.6) µm, (x̄ = 68 × 1.9 µm), fascicled, filiform, multi-septate, thin-walled, hyaline, rough with taper, obtuse ends, without oil guttules, hyaline, slightly smooth. ***Asexual morph***: Not observed.

**Figure 2.**
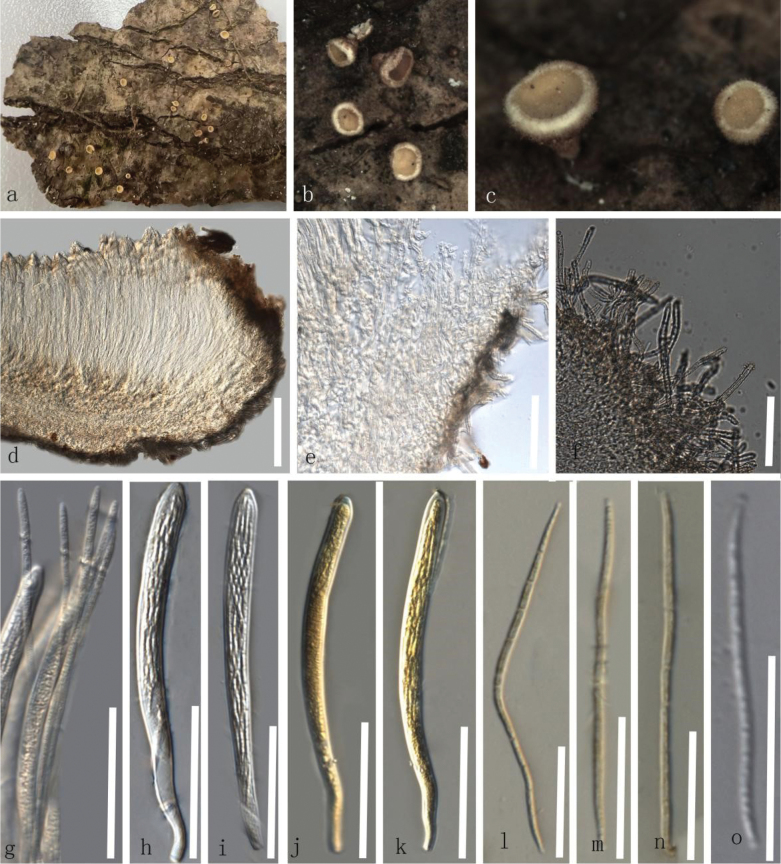
*Erioscyphellaailaoensis* (HKAS135686, holotype) **a–c** dried ascomata on the bark **d** vertical section of an ascoma **e** excipulum **f** hairs **g** paraphyses **h–k** asci (**j, k** asci in MLZ) **l–o** ascospores (**l–n** ascospores in MLZ). Scale bars: 100 µm (**d**); 50 µm (**e–k**); 30 µm (**l–o**).

##### Material examined.

China • Yunnan Province, Puer City, Jingdong County, Ailao Mountain, altitude 2478 m, on the decayed unidentified bark, 8 June 2022, Hongli Su, SU872 (HKAS135686, ***holotype***); China • Xizang Province, Shigatse City, altitude 1774 m, on the decayed unidentified twig, 6 July 2022, Hongli Su, SU1423 (HKAS135687, ***paratype***).

##### Notes.

Our specimens, HKAS135686 and HKAS135687, were grouped as a distinct clade, separated from the clade comprising *E.abnormis* and *E.sclerotii* by 73% MLBS and 0.89 BIPP (Fig. 1). The new species exhibited morphological differences from *E.abnormis* and *E.sclerotii* by having J- apical pores, whereas the latter species have apical pores that are J- in MLZ. In contrast to the septate paraphyses of *E.abnormis*, *E.ailaoensis* has aseptate paraphyses (Han et al. 2021). Furthermore, asci and ascospores of *E.ailaoensis* are longer than those of *E.sclerotii* (Nagao 1996; Perić and Baral 2014). Therefore, *E.ailaoensis* is introduced here as a new species.

#### ﻿ Erioscyphella baimana

Taxon classificationFungiHelotialesLachnaceae

L. Luo, K.D. Hyde & Q. Zhao sp. nov.

3990227C-959B-58C4-B102-458622D471C8

Index Fungorum: IF902530

Facesoffungi Number: FoF16391

Fig. 3

##### Etymology.

The epithet “*baimana*” refers to the collection site, Baima Mountain, where the holotype specimen was collected.

##### Holotype.

HKAS 135697.

##### Description.

***Saprobic*** on dead twigs. ***Sexual morph*: *Apothecia*** superficial, gregarious, 0.3–1.1 mm in diameter, 0.3–1.4 mm high when dry, discoid to cupulate, long stipitate, externally covered with short, white hairs. ***Disc*** concave, surface slightly smooth, yellow. ***Margin*** flat to slightly involute, white, covered with white hairs. ***Receptacle*** cupulate to discoid, white, covered entirely with short, white hairs. ***Stipe*** 0.2–0.6 mm in diameter, 0.3–1.1 mm long when dry, cylindrical, solitary, white, clothed with white hairs. ***Hairs*** 30–120 × 2.8–4.7 µm (x̄ = 74 × 3.7 µm, n = 30), clavate to cylindrical, straight to slightly curved, aseptate, hyaline, thin-walled, covered with fine granules, obtuse apex, lacks apical amorphous. ***Hymenium*** 165–230 µm (x̄ = 195 µm, n = 12), concave, surface slightly smooth, yellow in dry. ***Medullary excipulum*** 35–120 µm (x̄ = 70 µm, n = 18), thin, hyaline, thin-walled cells of ***textura porrecta***, 1.3–3.8 µm (x̄ = 2.5 µm, n = 50) in diameter. ***Ectal excipulum*** 40–120 µm (x̄ = 68 µm, n = 18) thin, thin-walled, smooth, light yellowish cells of ***textura porrecta*** to ***oblita***, 1.9–6.3 µm (x̄ = 4.1 µm, n = 60) in diameter. ***Paraphyses*** 95–170 × 1.2–2.5 µm (x̄ = 140 × 1.6 µm, n = 25), longer than asci, filiform, straight to slightly curved, aseptate, hyaline, light smooth, with slightly obtuse apex. ***Asci*** 100–152 × 3.6–9.5 µm (x̄ = 123 × 7.2 µm, n = 34), 8-spored, unitunicate, clavate, straight to slightly curved, inoperculate, hyaline, slightly smooth, with an apical, amyloid pore and rounded ends, J+ in MLZ, tapered long stipitate base. ***Ascospores*** (85/7/2) (29.5–)30.5–37.5(–40.0) × 2.2–5.0 (–5.5) µm, (x̄ = 32.9 × 4.1 µm, n = 85), biseriate, fusoid-clavate with blunt ends, fusiform, 1–3-septate, thin-walled, hyaline, slightly smooth, tapering towards the obtuse ends, with longitudinal striations, without oil guttules. ***Asexual morph***: Not observed.

**Figure 3.**
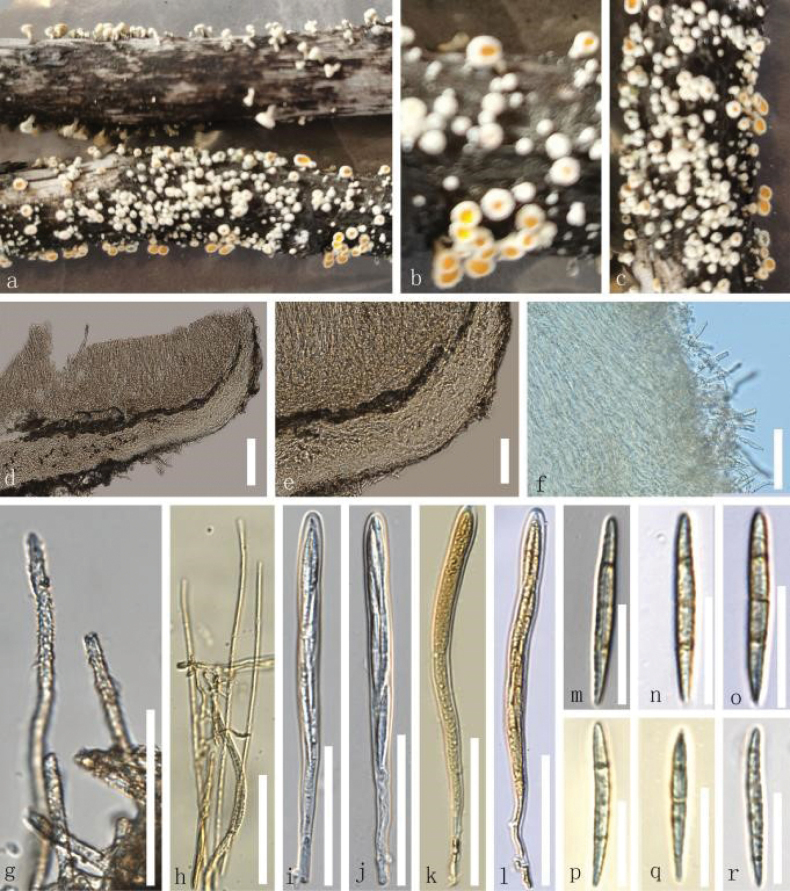
*Erioscyphellabaimana* (HKAS 135697, holotype) **a–c** dried ascomata on the twig **d** a vertical section of part of an ascoma **e** excipulum **f, g** hairs **h** paraphyses **i–l** asci (**k, l** asci in MLZ) **m–r** ascospores. Scale bars: 100 µm (**d–f**); 50 µm (**g–l**); 30 µm (**m–r**).

##### Material examined.

China • Yunnan Province, Diqing City, Deqin County, Baima Mountain, altitude 3485 m, on the decayed unidentified twig, 21 July 2022, Le Luo, Ly7 (HKAS 135697, ***holotype***); • *ibid*., Le Luo, Ly26 (HKAS 135696, ***isotype***).

##### Notes.

Our specimens, HKAS 135697 and HKAS 135696, were grouped in a distinct clade, separated from *E.latispora* by 99% MLBS and 1.00 BIPP (Fig. 1). Our species, *E.baimana*, morphologically differs from *E.latispora* by having aseptate hairs, ascospores without oil guttules whereas the latter species possess septate hairs, asci with rounded to subconical apex, and ascospores with four or more large guttules. Therefore, *E.baimana* is introduced here as a new species.

#### ﻿ Erioscyphella gelangheica

Taxon classificationFungiHelotialesLachnaceae

L. Luo, K.D. Hyde, H.L. Su & C.J.Y. Li sp. nov.

D23E127D-D20D-5551-9751-0FBBBFC813D3

Index Fungorum: IF902529

Facesoffungi Number: FoF16390

Fig. 4

##### Etymology.

The epithet “*gelangheica*” refers to the collection site Gelanghe township where the holotype specimen was collected.

##### Holotype.

HKAS135689.

##### Description.

***Saprobic*** on dead bark. ***Sexual morph*: *Apothecia*** scattered to partly gregarious, superficial, 0.24–0.5 mm in diameter, 0.35–0.5 mm high when dry, discoid to cupulate, long stipitate, externally covered with short, white to yellowish hairs. ***Discs*** concave, surface slightly rough, white to yellow. ***Margin*** flat to slightly involute, white to pale yellow, covered with white to pale yellow hairs. ***Receptacle*** discoid to cupulate, white to pale brown, clothed entirely with short, white to slightly yellow hairs. ***Stipe*** 0.06–0.18 mm in diameter, 0.14–0.3 mm long when dry, cylindrical, solitary, white to pale yellow, clothed with white to pale yellow hairs. ***Hairs*** 28–117 × 1.6–3.9 µm (x̄ = 60 × 2.9 µm, n = 30), clavate to cylindrical, straight to slightly curved, septate, hyaline, thin-walled, covered with hyaline granules, obtuse apex. ***Hymenium*** 65–120 µm (x̄ = 85 µm, n = 12), concave, surface slightly rough, light yellow in dry. ***Medullary excipulum*** 23.5–65 µm (x̄ = 37 µm, n = 18), thin, hyaline to light yellow, thin-walled cells of ***textura intricata***, 1.3–3.6 µm (x̄ = 2.2 µm, n = 50) in diameter. ***Ectal excipulum*** 20–95 µm (x̄ = 48 µm, n = 18) thick, thin-walled, smooth, hyaline cells of ***textura porrecta*** to ***textura globulosa***, 1.6–4.2 µm (x̄ = 2.7 µm, n = 60). ***Paraphyses*** 25–68 × 0.9–2.0 µm (x̄ = 43 × 1.3 µm, n = 25), longer than asci, filiform, straight, aseptate, hyaline, thin-walled, rough, with slightly acute apex. ***Asci*** 35–58 × 2.2–3.60 µm (x̄ = 46 × 3.0 µm, n = 34), 8-spored, unitunicate, clavate, straight to slightly curved, inoperculate, hyaline, wall apically thickened, laterally relatively thin, slightly smooth, with an apical, amyloid pore and tapered ends, J+ in MLZ. ***Ascospores*** (85/6/2) 6.0–8.3 × 1.2–1.8 µm, (x̄ = 7.3 × 1.5 µm, n = 86), partially biseriate, filiform, aseptate, thin-walled, hyaline, rough with tapering towards the obtuse ends, partially oil guttules, subspherical, hyaline, slightly smooth. ***Asexual morph***: Not observed.

**Figure 4.**
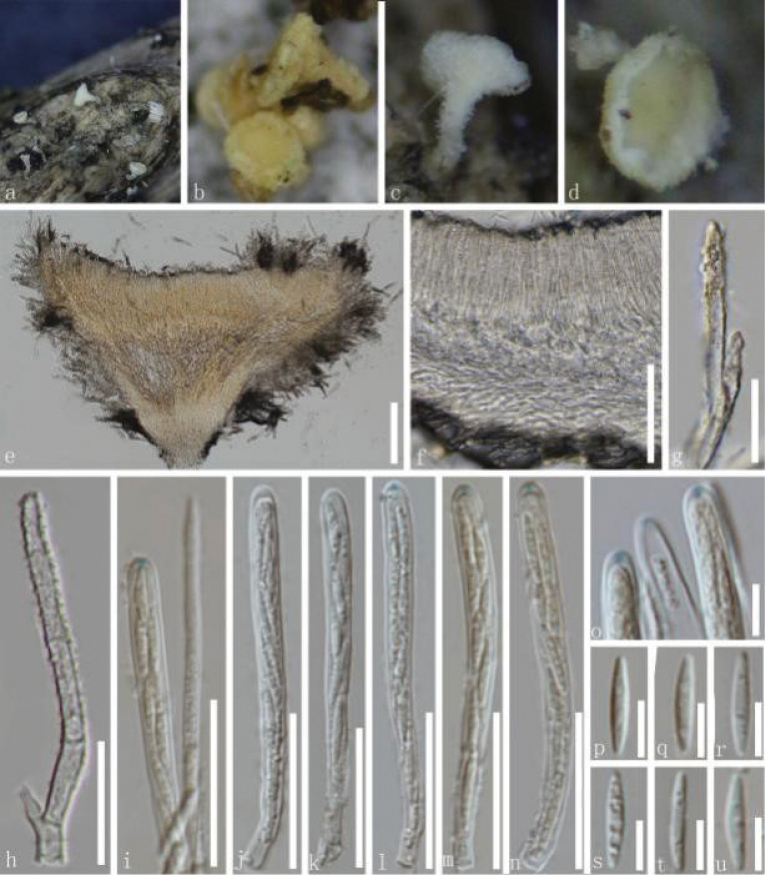
*Erioscyphellagelangheica* (HKAS 135689, holotype) **a–d** dried ascomata on the host **e** a vertical section of an ascoma **f** excipulum **g, h** hairs **i** paraphyses and asci **j–n** asci (**l–n** asci in MLZ) **o** apices of asci treated with Melzer’s reagent **p**–**u** ascospores. Scale bars: 100 µm (**e**); 50 µm (**f**); 20 µm (**g–n**); 5 µm (**o–u**).

##### Material examined.

China • Yunnan Province, Xishuangbanna City, Menghai County, Gelanghe township, altitude 2097 m, on the decayed unidentified bark, 6 September 2022, Cuijinyi Li, LCJY1389 (HKAS 135689, ***holotype***); • *ibid*., Hongli Su, SU1978 (HKAS 135695, ***paratype***).

##### Notes.

Our specimens, HKAS 135689 and HKAS 135695, were grouped in a distinct clade, separated from the clade comprising *E.abnormis*, *E.sclerotii*, *E.ailaoensis*, *E.brasiliensis* and *E.aseptata* by 78% MLBS and 0.88 BIPP (Fig. 1). *Erioscyphellagelangheica* has shorter asci (35–58 µm vs. 41–104 µm), ascospores (6.0–8.3 µm vs. 39–81 µm), and paraphyses (25–68 µm vs. 52–123 µm) than those of *E.abnormis* (Perić and Baral 2014). *Erioscyphellagelangheica* differs from *E.sclerotii* by having long stipitate apothecia and aseptate ascospores, while *E.sclerotii* has short stipitate apothecia and 1–3-septate ascospores. Our species, *E.gelangheica*, has shorter asci (35–58 µm vs. 85–143 µm), ascospores (6.0–8.3 µm vs. 45.5–97 µm), and paraphyses (25–68 µm vs. 100–138 µm) than those of *E.ailaoensis*. Furthermore, *E.ailaoensis* has septate ascospores in contrast to the aseptate ascospores of *E.gelangheica*. Compared to long stipitate apothecia with white to pale yellow hairs and aseptate ascospores of *E.gelangheica*, *E.brasiliensis* has 0–1-septate ascospores and stipitate apothecia, with the stipe base often devoid of hairs and blue-black (Haines 1992). In addition to having aseptate ascospores, *E.gelangheica* has shorter asci (35–58 µm vs. 70–100 µm) and ascospores (6.0–8.3 µm vs. 28.5–45.6 µm) than those of *E.aseptata*, which has septate ascospores (Ekanayaka et al. 2019). Therefore, *E.gelangheica* is introduced here as a new species.

#### ﻿ Erioscyphella tengyueica

Taxon classificationFungiHelotialesLachnaceae

L. Luo, K.D. Hyde & C.J.Y. Li sp. nov.

DB27D074-1CBF-57D6-8D64-62275C92C5D3

Index Fungorum: IF902531

Facesoffungi Number: FoF16392

Fig. 5

##### Etymology.

The epithet refers to the collection site of the type specimen.

##### Holotype.

HKAS 135688.

##### Description.

***Saprobic*** on the dead twigs. ***Sexual morph*: *Apothecia*** superficial, scattered to partly gregarious, 0.16–0.68 mm in diameter, 0.3–0.7 mm high when dry, discoid to cupulate, shortly stipitate, externally covered with short, white hairs. ***Discs*** concave, surface slightly rough, white. ***Margin*** slightly involute, white, covered with white hairs. ***Receptacle*** cupulate, concolorous, clothed entirely with short, white hairs. ***Stipe*** 0.09–0.23 mm in diameter, 0.18–0.4 mm long when dry, cylindrical, solitary, concolorous with the receptacle, clothed with white hairs. Hairs 45–95 × 2.8–6.9 µm (x̄ = 68 × 4.8 µm, n = 10), clavate to cylindrical, straight or curved, septate, hyaline, thin-walled, less covered with hyaline granules, obtuse apex. ***Hymenium*** 65–115 µm (x̄ = 87 µm, n = 12), concave, surface slightly rough, light white in dry. ***Medullary excipulum*** 18–33 µm (x̄ = 26 µm, n = 18), thick, comprising hyaline, thin-walled, poorly developed cells of ***textura globulosa***, 1.4–3.5 µm (x̄ = 2.4 µm, n = 50) in diameter. Ectal excipulum 11–30 µm (x̄ = 21 µm, n = 18) thick, comprising thick-walled, smooth, light yellowish cells of ***textura oblita*** to ***textura porrecta***, 1.2–3.8 µm (x̄ = 2.2 µm, n = 60). ***Paraphyses*** 65–97 × 1.8–3.0 µm (x̄ = 86 × 2.5 µm, n = 25), longer than asci, filiform, straight, aseptate, hyaline, thin-walled, narrow lanceolate, smooth, less covered with hyaline granules, with slightly obtuse apex. ***Asci*** 60–80 × 6.0–9.3 µm (x̄ = 70 × 7.6 µm, n = 34), 8-spored, clavate, straight to slightly curved, inoperculate, hyaline, unitunicate, slightly smooth, with an apical, amyloid pore and rounded ends, croziers absent at the basal septum, J+ in MLZ. ***Ascospores*** (80/6/2) 25–31.5 × 1.6–5.5 µm, (x̄ = 27.7 × 3.3 µm, n = 80), overlapping biseriate, filiform, aseptate, thin-walled, hyaline, rough with tapering towards obtuse ends, filled with oil guttules or with 1–2- large oil guttules. ***Asexual morph***: Not observed.

**Figure 5.**
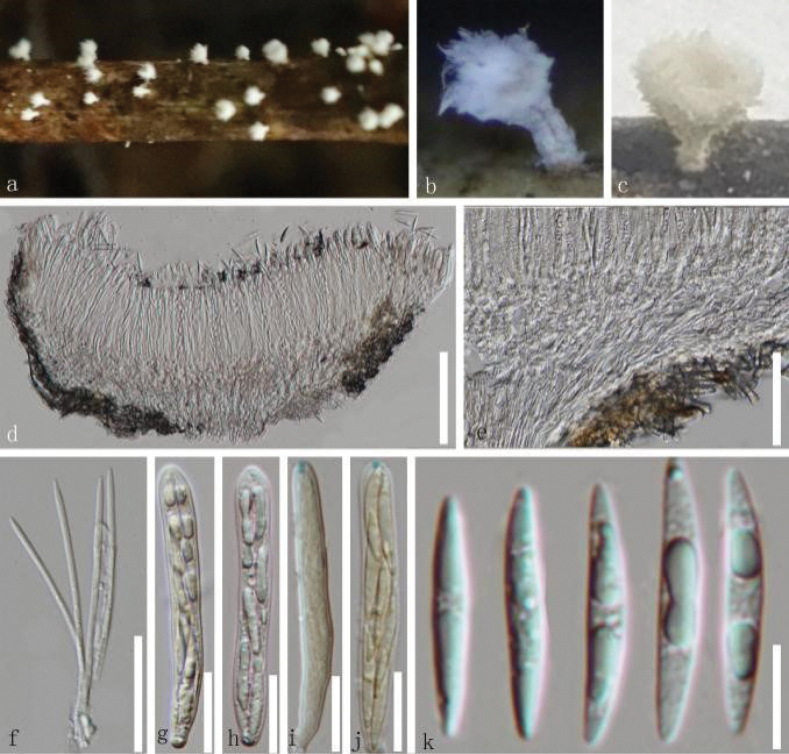
*Erioscyphellatengyueica* (HKAS 135688, holotype) **a–c** dried ascomata on the host **d** a vertical section of an ascoma **e** excipulum **f** paraphyses **g–j** asci (**i, j** asci in MLZ) **k** ascospores. Scale bars: 100 µm (**d**); 50 µm (**e, f**); 20 µm (**g–j**); 10 µm (**k**) .

##### Material examined.

China • Yunnan Province, Tengchong City, Tengyue Street, altitude 1983.3 m, on the decayed unidentified twig, 21 August 2022, Cuijinyi Li, LCJY1171 (HKAS 135688, ***holotype***); • *ibid*., altitude 1774 m, on the decayed unidentified twig, 18 August 2022, Le Luo, Ly255 (HKAS 135693, ***paratype***).

##### Notes.

Our specimens, HKAS 135688 and HKAS 135693, were grouped into a distinct clade, separated from *E.papillaris* (TNS-F-81272) by 91% MLBS and 0.98 BIPP (Fig. 1). *Erioscyphellatengyueica* differs from *E.papillaris* by having aseptate ascospores and aseptate paraphyses, while the latter has septate paraphyses and aseptate or one-septate (rarely two-septate) ascospores. Our species differs from *E.otanii* by having longer asci (60–80 µm vs. 34–38.8 µm), longer ascospores (34–38.8 µm vs. 12.3–14.6 µm) and aseptate paraphyses, in contrast to the septate paraphyses of *E.otanii* (Tochihara and Hosoya 2022). Therefore, *E.tengyueica* is introduced here as a new species.

## ﻿Discussion

In China, the diversity of Leotiomycetes is substantial due to varied climates and ecosystems in the country (Li et al. 2022; Su et al. 2023; Guo et al. 2024; Luo et al. 2024; Zhang et al. 2024). Ongoing research continues to uncover new species and understand their roles in ecosystem functioning, highlighting the importance of preserving fungal diversity for ecological health and agricultural sustainability (Li et al. 2022; Su et al. 2023; Lestari and Chethana 2024; Luo et al. 2024).

Based on the previous research on the phylogeny, morphology and ecology of Lachnaceae (Hosoya et al. 2010), delimiting generic boundaries in Lachnaceae can no longer be defined by morphological characteristics alone (Li et al. 2022; Tochihara and Hosoya 2022; Su et al. 2023). The latest concept for *Erioscyphella* was proposed as the typical taxa characterized by the hair structures (the swollen apices, apical anamorph material/resinous material) and ascospore length (Tochihara and Hosoya 2022). This revision signifies a departure from the previously emphasized characteristics, highlighting the dynamic nature of fungal taxonomy (Tochihara and Hosoya 2022).

Despite advances in molecular techniques and the integration of morphological and ecological data, our analysis reveals persistent challenges in clarifying species boundaries. Particularly for paraphyletic members of long-spored *Lachnum* within *Erioscyphella*, the utilization of the UNITE Species Hypotheses (SH) system analysis based on ITS gene fragment, alongside traditional methods, has not provided definitive resolutions, indicating the ongoing complexity in species delineation (Tochihara and Hosoya 2022). Phylogenetic relationships in *Erioscyphella* gradually became clear as more fresh collections were identified with sequence data (Li et al. 2022; Su et al. 2023). Almost all species of *Erioscyphella* have stable phylogenetic positions with strong statistical support, except for *E.bambusina*, which lacks sequences in public databases.

Nearly all *Erioscyphella* species occupy stable phylogenetic positions supported by strong statistical evidence, except for *E.bambusina*, which lacks sequence data in public databases. We compared the morphology of *E.bambusina* with our newly established species (Dennis 1954; Su et al. 2023). *Erioscyphellabambusina* has septate, shorter ascospores, and septate paraphyses, in contrast to the multi-septate, longer ascospores, and aseptate paraphyses of *E.ailaoensis*. *Erioscyphellagelangheica* differs from *E.bambusina* by having smaller apothecia and aseptate paraphyses, while the latter has larger apothecia and septate paraphyses. *Erioscyphellabaimana* has aseptate hairs and aseptate paraphyses, while *E.tengyueica* has white discs, and J- in MLZ with and without 3% KOH pretreatment. In contrast to these two, *E.bambusina* has cream to pale yellow discs, pore blued in MLZ, and septate paraphyses.

Most records of *Erioscyphella* are from the tropics (Spooner 1987; Haines and Dumont 1983; Tochihara and Hosoya 2022). Although sample collections have extended to subtropic, temperate and cold-temperate regions (Bien and Damm 2020; Tochihara and Hosoya 2022), collected samples are still scarce. Further, it was found that the lack of available sequences of these species led to Lachnaceae and Helotiaceae taxa exhibiting paraphyletic characters in the ITS-LSU phylogeny (Johnston et al. 2019; Quandt and Haelewaters 2021). Although Johnston et al. (2019) solved this issue based on phylogenetic analyses of up to 15 concatenated genes across 279 specimens, obtaining gene sequences for multiple loci for Lachnaceae taxa is still one of the critical problems.

Continued interdisciplinary research efforts are warranted to refine our understanding of fungal taxonomy within the Lachnaceae. Future studies should explore novel methodologies, such as high-throughput sequencing and ecological niche modeling, to elucidate species boundaries and evolutionary relationships more comprehensively. Additionally, comprehensive taxonomic revisions, including detailed examination of type specimens and expanded sampling, will be crucial for resolving taxonomic ambiguities and advancing fungal systematics.

In conclusion, our study contributes to the growing body of knowledge on fungal taxonomy and highlights the need for an integrated approach combining molecular, morphological, and ecological data to address the complexities inherent in delineating generic boundaries and species relationships within the Lachnaceae family.

## Supplementary Material

XML Treatment for Erioscyphella ailaoensis

XML Treatment for Erioscyphella baimana

XML Treatment for Erioscyphella gelangheica

XML Treatment for Erioscyphella tengyueica

## ﻿Additional information

### Author ORCIDs

Le Luo https://orcid.org/0009-0006-7213-0498

Kandawatte W. Thilini Chethana https://orcid.org/0000-0002-5816-9269

Qi Zhao https://orcid.org/0000-0001-8169-0573

Hong-Li Su https://orcid.org/0000-0001-9071-7635

Cui-Jin-Yi Li https://orcid.org/0000-0002-2805-7071

Vinodhini Thiyagaraja https://orcid.org/0000-0002-8091-4579

Fatimah Al-Otibi https://orcid.org/0000-0003-362

Kevin D. Hyde https://orcid.org/0000-0002-2191-0762

### Data availability

All of the data that support the findings of this study are available in the main text.
